# Targeting Polyamine Metabolism in Colorectal Cancer: Apigenin Dismantles the HIF-1α/SMOX Positive Feedback Loop to Suppress Tumor Progression

**DOI:** 10.3390/ijms27073261

**Published:** 2026-04-03

**Authors:** Zhengkun Zhang, Bin Xiang, Ruiman Geng, Xuxu Ji, Dingxue Wang, Zhaoru Yin, Lihong Chen, Ji Liu

**Affiliations:** 1 Department of Biochemistry & Molecular Biology, West China School of Basic Medical Sciences & Forensic Medicine, Sichuan University, Chengdu 610041, China; zzkbrucee@gmail.com (Z.Z.);; 2 Chongqing Advanced Pathology Research Institute, Jinfeng Laboratory, Chongqing 401329, China

**Keywords:** apigenin, colorectal cancer, inflammatory, SMOX

## Abstract

Tumor microenvironments, particularly hypoxia and inflammation, heavily influence colorectal cancer (CRC) pathogenesis by altering polyamine metabolism. Identifying natural compounds targeting these vulnerabilities remains critical. Integrating untargeted metabolomics, network pharmacology, and a human endogenous metabolite library screen, we identified apigenin (API) as a potent anti-CRC candidate. API significantly inhibited the proliferation, migration, and invasion of RKO and HCT116 cells in vitro and suppressed xenograft tumor growth in vivo. Crucially, high-throughput screening revealed that polyamines rescued CRC cells from API-induced cytotoxicity. Mechanistically, API exerts its effects by dismantling a newly identified HIF-1α/SMOX positive feedback loop. In CRC, HIF-1α transcriptionally activates spermine oxidase (SMOX), while SMOX-driven polyamine metabolism fuels the TLR4/MyD88 inflammatory cascade to continuously stabilize HIF-1α. API acts as a “circuit breaker” for this axis, significantly reducing the spermidine/spermine ratio and downregulating inflammatory signaling. Ultimately, API effectively remodels polyamine metabolism and suppresses CRC progression by disrupting the HIF-1α/SMOX and TLR4/MyD88 pathways, offering a novel metabolic mechanism for API in CRC therapy.

## 1. Introduction

Colorectal cancer (CRC) is a malignant tumor that occurs in the colon and rectum. CRC ranks third in morbidity and second in mortality among malignant tumors worldwide [1]. The highest incidence of CRC is the rectum. CRC is affected by genetic, environmental and lifestyle factors such as other cancers including microbial disorders, obesity, type II diabetes and other diseases [2]. It is worth noting that the age of onset of CRC is getting younger. The median age of diagnosis of colorectal cancer is 65 years old for men and 68 years old for women. Since the beginning of the 21st century, the incidence of adults under 50 years old has increased significantly, while the incidence of the elderly population has declined [3]. At present, the cause of the younger onset of CRC is not fully determined, which may be related to changes in diet and lifestyle. Studies have found that a healthy diet is also very important for the prevention of CRC. Diets rich in grains or dietary fiber can regulate the level of microorganisms in the body and further reduce the risk of CRC [4,5]. In recent years, the diagnosis and treatment of CRC have achieved certain development results, but many patients still face the risk of postoperative recurrence and metastasis. Therefore, it is particularly important to explore the molecular biological characteristics of CRC and its specific pathogenesis.

Studies have shown that lipopolysaccharide (LPS) concentrations are elevated in tumor tissues of colorectal cancer patients. Intestinal barrier dysfunction is accompanied by increased intestinal permeability, which causes bacteria or their cell wall components such as LPS to be transferred to the blood, leading to persistent systemic inflammation and aggravating CRC damage [6]. Different regulatory strategies for gut microbiota and their metabolites, including healthy diet, probiotics and microbiota transplantation, can prevent the occurrence and progression of CRC [7,8]. Polyamine (PA) is a kind of aliphatic amine small molecule compound containing multiple amino groups, which is widely found in organisms, including putrescine (Put), spermidine (Spd) and spermine (Spm). They are involved in various cellular functions, including protein synthesis, DNA and RNA structures, protein RNA interactions, and gene expression [9,10,11]. Studies have shown that the stability of polyamine levels in normal cells is regulated by polyamine metabolism, but, in many types of cancer, polyamine metabolism is disordered and, ultimately, polyamine metabolism imbalance leads to cell damage and tissue damage [12]. Polyamine levels are involved in the regulation of tumor microenvironment and are significantly increased during tumor development. Therefore, the regulation of polyamine metabolism is one of the important strategies for anti-tumor therapy [13,14].

The level of polyamines in the body’s cells is very important for maintaining its homeostasis. The homeostasis of polyamine levels is regulated by various pathways such as polyamine biosynthesis, catabolism, and polyamine transport pathways. A variety of enzymes are involved in the metabolic pathway of polyamines. Each of these enzymes may change the homeostasis of polyamines to respond to a variety of cellular signals and produce reactive oxygen species and aldehydes. Each product may cause the body to become ill [15]. Spermine oxidase (SMOX) is a key enzyme involved in polyamine catabolism. It can use Spm as a substrate and oxidize it to Spd to produce H_2_O_2_ and amino propionaldehyde [16]. SMOX can be over-activated under inflammatory stimulation, producing toxic substances, causing increased reactive oxygen species (ROS) and DNA damage to further promote the development of CRC [17,18]. SMOX plays a role in carcinogenesis, which may affect the interaction between infection, inflammation and carcinogenesis. The high expression of SMOX under various inflammatory stimuli, its ability to produce mutagenic ROS and its ability to destroy DNA indicate that SMOX may be an effective target for chemoprevention [19]. The development and application of targeted SMOX inhibitors play an important role in the prevention and treatment of related diseases. Therefore, exploring the effect of SMOX on the interaction between inflammation and tumor will provide a good idea for its preventive intervention.

Chemical resistance is a major limitation during cancer treatment. Tumors acquire drug resistance, resulting in the loss of therapeutic effect of frequently used chemotherapy drugs [20]. Natural medicines play an important role in the prevention and treatment of tumors. Studies have found that some natural products, which do not induce drug resistance, play an important role in regulating polyamine metabolism [21]. Therefore, the development of natural drugs is particularly important for the prevention and treatment of tumors. Apigenin (API) is a common natural flavonoid small molecule compound, which is rich in some Chinese herbal medicines and daily edible vegetables and fruits. It has a wide range of anti-tumor effects, and its pharmacological mechanism has attracted more attention [22,23]. In recent years, studies on the anti-tumor effect of apigenin have shown that the effect of apigenin on tumor cells is multifaceted, but most of the studies are not deep enough [23,24].

Recently, the integration of multi-omics and network pharmacology has provided powerful tools for elucidating the complex biological characteristics of CRC and discovering novel natural therapeutics. Metabolic reprograming is a hallmark of cancer, and untargeted metabolomics can systematically map the dynamic alterations of endogenous metabolites in the tumor microenvironment. Meanwhile, high-throughput metabolite library screening offers a cutting-edge approach to functionally pinpoint the exact metabolic nodes modulated by pharmacological interventions.

In the present study, we adopted a discovery-driven strategy. Initially, untargeted metabolomics and network pharmacology were integrated to highlight API and the HIF-1 signaling pathway. Following the validation of API’s anti-tumor phenotypes in vitro and in vivo, we pioneeringly employed a high-throughput endogenous metabolite library screening, which unexpectedly anchored our focus on polyamine metabolism and its key enzyme, SMOX. Crucially, we investigated the crosstalk between SMOX and HIF-1α, uncovering a mutually amplifying “positive feedback loop” that drives CRC progression. We comprehensively elucidated how API acts as a circuit breaker to dismantle this HIF-1α/SMOX axis and suppress the downstream TLR4/MyD88 inflammatory cascade, providing a solid theoretical basis for its anti-CRC effect.

## 2. Results

### 2.1. Metabolic Reprograming in Colorectal Cancer Revealed by Untargeted Metabolomics

**Figure 1:**
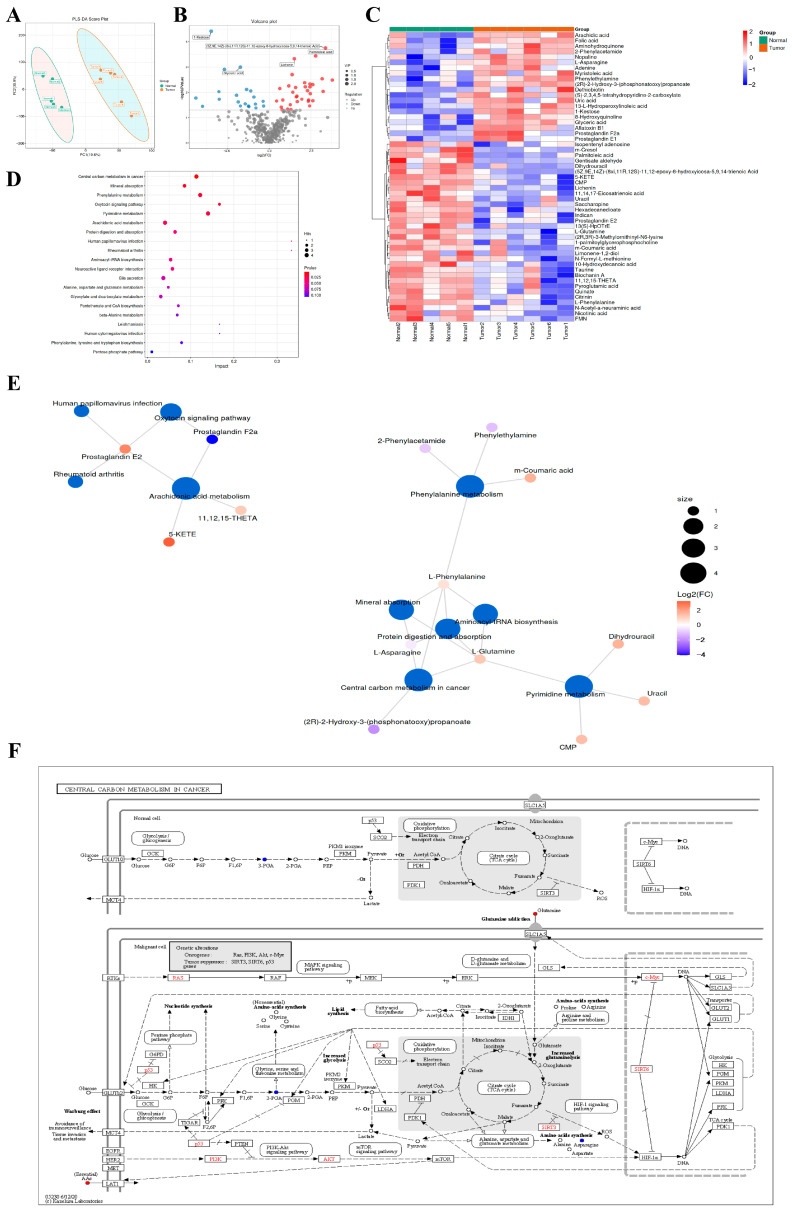
Untargeted metabolomics profiling reveals altered metabolic pathways in CRC. (**A**) Partial least squares discriminant analysis (PLS-DA) score plot of the Tumor and Normal groups. (**B**) Volcano plot highlighting significantly upregulated and downregulated metabolites, the horizontal dashed line represents the significance threshold of *p* < 0.05. (**C**) Heatmap visualization of differential metabolites. (**D**) Bubble chart of KEGG pathway enrichment analysis based on the altered metabolites. (**E**) Network visualization of the enriched metabolic pathways. (**F**) KEGG map illustrating the alterations in central carbon metabolism in cancer.

To elucidate the metabolic alterations associated with CRC, untargeted metabolomics was performed on 11 paired clinical specimens to compare tumor tissues with >5 cm normal adjacent tissues. The Partial least squares discriminant analysis (PLS-DA) score plot demonstrated a clear separation between the tumor and normal groups (Figure 1A), indicating significant metabolic reprogramming. The volcano plot highlighted the differentially expressed metabolites (Figure 1B). Further Kyoto Encyclopedia of Genes and Genomes (KEGG) pathway enrichment analysis revealed that these metabolic alterations were predominantly enriched in arachidonic acid metabolism, central carbon metabolism in cancer, and aminoacyl-tRNA biosynthesis (Figure 1D–F). These findings suggest that targeting inflammatory metabolic pathways and central carbon metabolism could be a promising strategy for CRC therapy.

### 2.2. Network Pharmacology Identifies Apigenin as a Potential Therapeutic Agent

**Figure 2:**
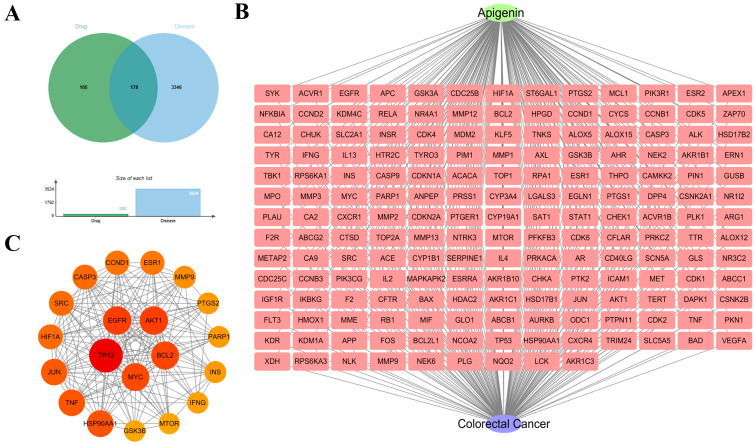
Network pharmacology analysis identifies apigenin targets and related pathways in CRC. (**A**) Venn diagram showing the intersection between drug targets and CRC-related targets. (**B**) The compound–target-disease network for apigenin. (**C**) Hub genes identified within the core protein–protein interaction (PPI) network, node colors ranging from dark red to yellow and their decreasing sizes indicate the descending order of the targets’ topological importance.

**Figure 3:**
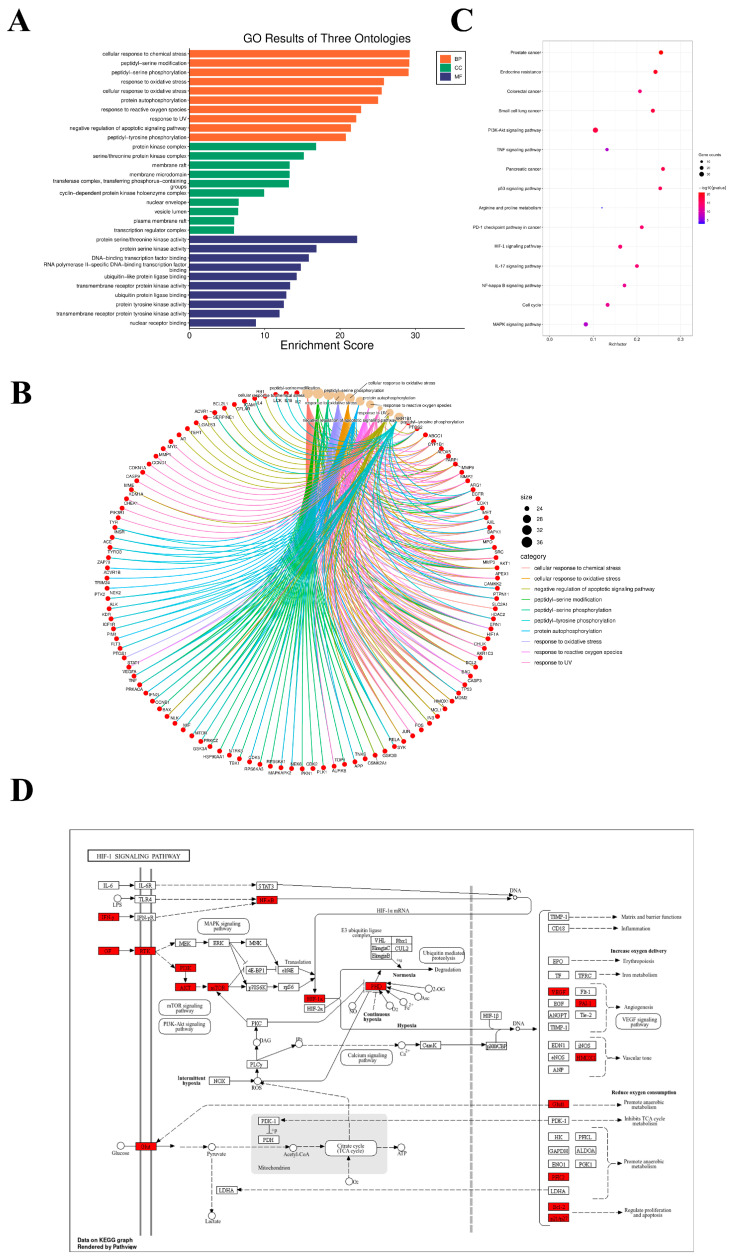
(**A**) Gene Ontology (GO) enrichment analysis, including Biological Process (BP), Cellular Component (CC), and Molecular Function (MF), highlighting protein serine/threonine kinase activity. (**B**) Chord diagrams mapping the relationships between genes and enriched GO terms. (**C**) Bubble chart of KEGG pathway enrichment analysis. (**D**) Detailed KEGG map of the HIF-1 signaling pathway highlighted by the analysis. The red color highlights the identified target genes, solid and dashed lines indicate direct and indirect interactions respectively, and arrows denote the direction of activation within the pathway.

Given the observed inflammatory and metabolic abnormalities, we employed network pharmacology to identify potential natural candidates. The Venn diagram illustrated the intersection of drug targets and CRC-related disease targets (Figure 2A). By analyzing the protein–protein interaction (PPI) network, we identified key hub genes, notably including *HIF1A, MYC,* and *TP53* (Figure 2B,C). Subsequent Gene Ontology (GO) and KEGG enrichment analyses indicated that these targets are highly associated with protein serine/threonine kinase activity and the HIF-1 signaling pathway (Figure 3A–D). Based on these predictions, API emerged as a prime candidate for further investigation.

### 2.3. API Inhibits Proliferation, Migration, and Invasion and Promotes Apoptosis of CRC Cells

**Figure 4:**
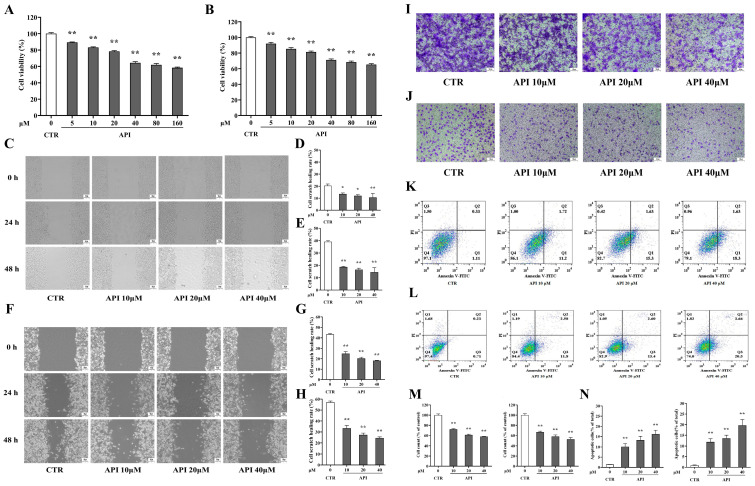
API inhibits proliferation, migration, and invasion and promotes apoptosis of colorectal cancer cells in vitro. (**A**,**B**) Cell viability of RKO and HCT116 cells treated with varying concentrations of API evaluated by CCK-8 assay. (**C**–**H**) Cell scratch assays and quantitative analysis showing the migration capability of RKO and HCT116 cells at 0, 24, and 48 h. Scale bar = 100 μm. (**I**,**J**,**M**,**N**) Transwell invasion assays and cell counts demonstrating the invasive capacity of the cells. Scale bar = 100 μm. (**K**,**L**) Cell apoptosis detected by Annexin V-FITC/PI flow cytometry. * *p* < 0.05 and ** *p* < 0.01 compared to the control group. Scale bar = 100 μm.

The effect of API (5–160 μM) on RKO and HCT116 cell viability was evaluated using a CCK8 assay after 24 h of incubation. As shown in Figure 4A,B, API significantly suppressed the cell survival rate in both RKO and HCT116 cells in a dose-dependent manner. Thus, 10, 20, and 40 μM of API were used in subsequent experiments. Furthermore, cell scratch and Transwell invasion assays were performed to evaluate cell motility. The results demonstrated that RKO and HCT116 cells treated with API exhibited significantly lower wound healing rates (Figure 4C–H) and decreased invasiveness (Figure 4I,J,M,N) compared to control cells. Finally, flow cytometry analysis revealed that the proportion of apoptotic cells in the API-treated groups was significantly higher than that in the control group (Figure 4K,L). Together, these studies indicate that API exerts a potent anti-tumor role by inhibiting the proliferation, migration, and invasion, while promoting the apoptosis of CRC cells.

### 2.4. API Suppresses Colorectal Tumor Growth In Vivo

**Figure 5:**
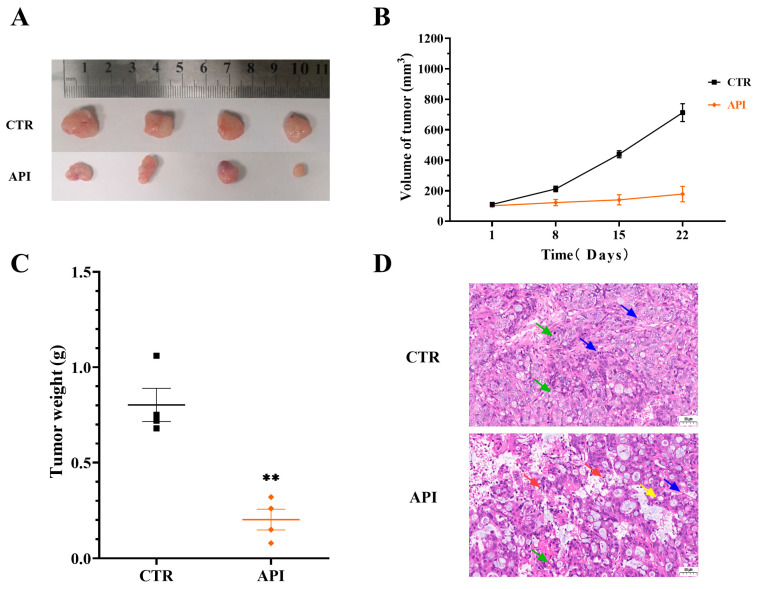
API inhibits xenograft tumor growth in nude mice. (**A**) Representative images of isolated tumors from the control (CTR) and API-treated groups. (**B**) Tumor volume growth curves measured over 22 days. (**C**) Comparison of tumor weight at the experimental endpoint. ** *p* < 0.01 compared to the CTR group. (**D**) Representative H&E staining of tumor tissues indicating structural necrosis and inflammatory cell infiltration. Red arrows indicate apoptotic cells with fragmented or pyknotic nuclei and eosinophilic cytoplasm; green arrows denote mitotic figures or viable tumor cells; blue arrows show infiltration of inflammatory cells; and yellow arrows highlight cytoplasmic vacuoles. Scale bar = 50 μm.

At the end of the mouse xenograft experiment (3 weeks), the tumors were isolated. Tumor size (Figure 5A), volume (Figure 5B), and weight (Figure 5C) were significantly reduced by API treatment compared to the control (CTR) group. Furthermore, histological evaluation via H&E staining (Figure 5D) showed that tumor cells in the API group were loosely arranged with cytoplasmic vacuolization and significant inflammatory cell infiltration, accompanied by increased areas of tumor necrosis. Conversely, the CTR group displayed vigorously growing tumor cells with obvious atypia. These results further support the robust in vivo efficacy of API against CRC tumor growth.

### 2.5. High-Throughput Metabolite Library Screening Identifies Polyamine Metabolism and SMOX as Key Targets of API

**Figure 6:**
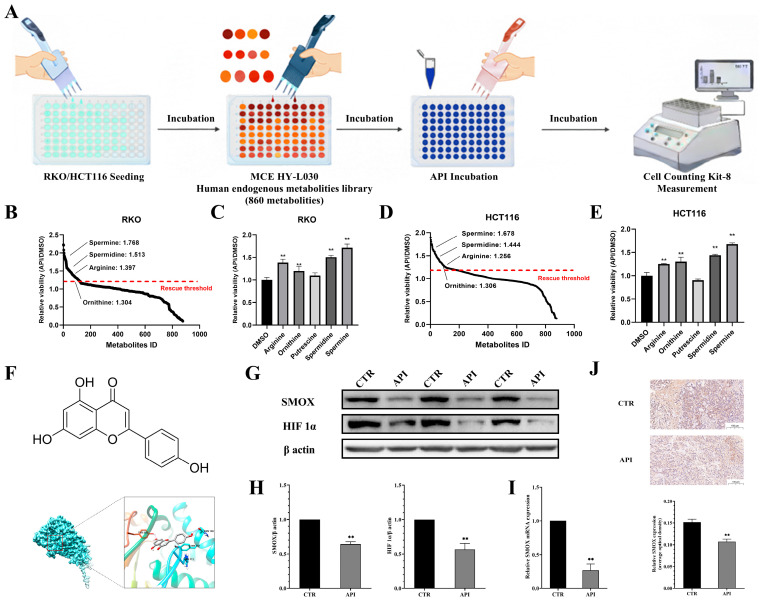
Metabolite library screening identifies polyamine metabolism and SMOX as crucial targets of API. (**A**) Schematic illustration of the MCE human endogenous metabolite library (860 metabolites) screening process via co-incubation with API. (**B**,**D**) Scatter plots ranking the relative viability (API/DMSO) of RKO and HCT116 cells. The red dashed line represents the strict empirical threshold for significant rescue (relative viability ratio ≥ 1.20). (**C**,**E**) Bar graphs validating the significant modulatory effects of specific polyamine-related metabolites (Arginine, Ornithine, Putrescine, Spermidine, and Spermine) on API efficacy. ** *p* < 0.01 compared to the DMSO group. (**F**) Molecular docking visualization displaying the binding conformation and interaction sites between API and SMOX. (**G**–**J**) Western blot, qRT-PCR, and immunohistochemistry (IHC) analyses validating the downregulation of SMOX and HIF-1α expression by API in xenograft tumor tissues. Scale bar = 100 μm. ** *p* < 0.01 compared to the CTR group.

While the phenotypic effects of API were evident, its precise metabolic targets remained unclear. To systematically decode this, we performed a high-throughput functional screening using an MCE human endogenous metabolite library comprising 860 metabolites (Figure 6A). By co-incubating these metabolites with API in RKO and HCT116 cells, we assessed their impact on API-induced cytotoxicity. Strikingly, the relative viability analysis revealed that polyamine-related metabolites—specifically spermine, spermidine, arginine, and ornithine—were the most significant modulators, rescuing CRC cells from API-induced cytotoxicity (Figure 6B–E). Given the central role of these metabolites, we directed our focus towards spermine oxidase (SMOX), a key enzyme in polyamine catabolism. Molecular docking analysis indicated a strong binding affinity (−8.553 kcal/mol) between API and SMOX (Figure 6F). Furthermore, in vivo validation confirmed that API significantly downregulated the expression of SMOX and the network-predicted downstream target, HIF-1α, in xenograft tumor tissues (Figure 6G–J). To validate that the anti-tumor effects of API are dependent on SMOX suppression, we further evaluated the cellular response using SMOX-overexpressing (SMOXOE) cell models. As expected, the introduction of exogenous SMOX rescued CRC cells from API-induced anti-proliferative and anti-migratory effects (Supplementary Figure S1). These functional rescue data establish SMOX as the primary metabolic target mediating API’s efficacy in CRC.

### 2.6. API Modulates the TLR4/MyD88 and HIF-1α Signaling Pathways

**Figure 7:**
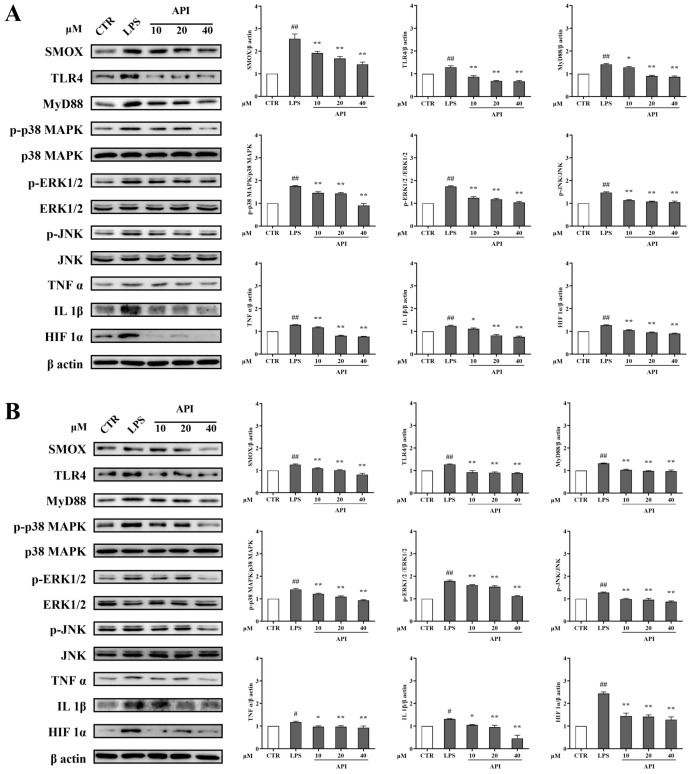
API modulates the TLR4/MyD88/HIF-1α inflammatory signaling pathways in CRC cells. (**A**) Western blot images and corresponding quantitative analysis of SMOX, TLR4, MyD88, p-p38 MAPK, p-ERK1/2, p-JNK, TNFα, IL-1β, and HIF-1α protein expression levels in LPS-stimulated RKO cells treated with API. (**B**) The corresponding Western blot analyses in HCT116 cells. # *p* < 0.05 and ## *p* < 0.01 compared to the CTR group, * *p* < 0.05 and ** *p* < 0.01 compared to the LPS group.

To investigate the anti-CRC effect of API through inflammation-related signaling pathways, the protein expression levels of SMOX, TLR4, MyD88, p-p38 MAPK, p-ERK1/2, p-JNK, TNFα, IL-1β, and HIF-1α were measured. As shown in Figure 7A (RKO cells) and Figure 7B (HCT116 cells), following LPS intervention, the expression levels of these pro-inflammatory and signaling proteins were significantly upregulated. Notably, API treatment effectively and dose-dependently reversed these LPS-induced upregulations. These results indicate that API exerts its anti-CRC effects by suppressing SMOX and downregulating the TLR4/MyD88 and downstream MAPK/ERK/HIF-1α inflammatory signaling cascades.

### 2.7. API Regulates Intracellular Polyamine Contents in CRC Cells

**Figure 8:**
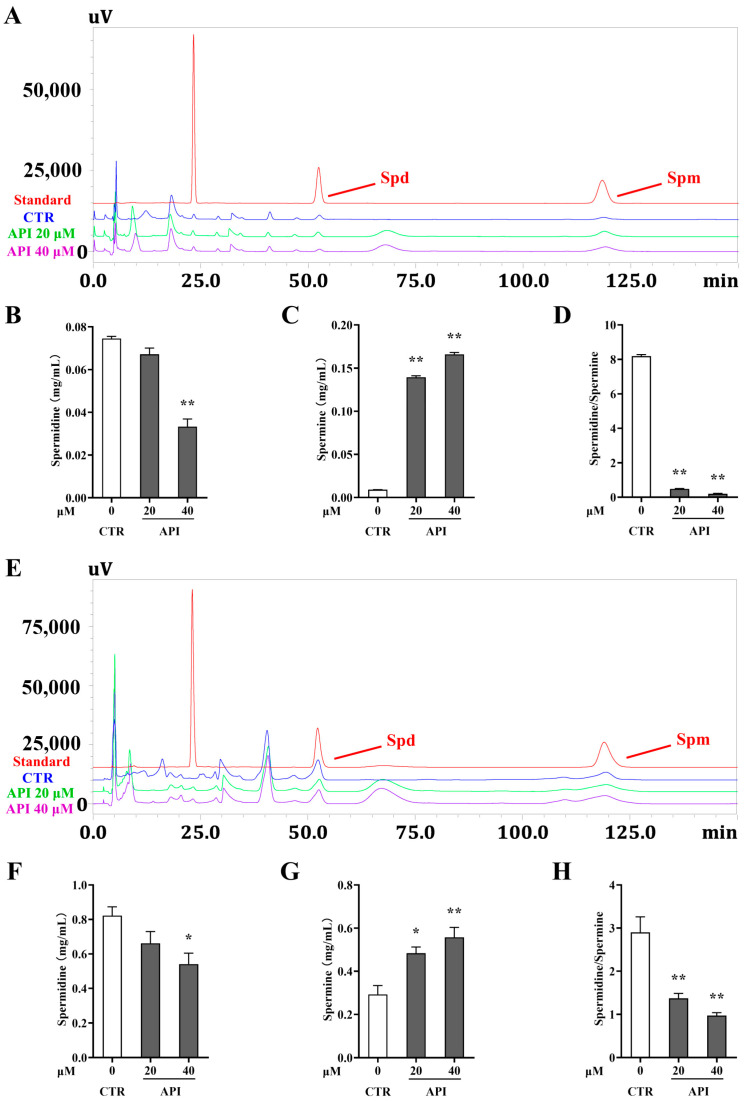
API regulates the content of polyamines in CRC cells. (**A**) The HPLC chromatograms of Spd and Spm in RKO cells. (**B**–**D**) The contents of Spd and Spm and the ratio of Spd to Spm in RKO cells. (**E**) The HPLC chromatograms of Spd and Spm in HCT116 cells. (**F**–**H**) The contents of Spd and Spm and the ratio of Spd to Spm in HCT116 cells. * *p* < 0.05, ** *p* < 0.01 vs. the LPS group.

Polyamines are a class of small molecular compounds widely present in organisms. SMOX is a key enzyme involved in the catabolism of polyamines. Spm can be oxidized to Spd under its action, producing H_2_O_2_ and amino propionaldehyde. In order to investigate the effect of API on polyamine metabolism, the contents of polyamines (Spd and Spm) in RKO and HCT116 cells were detected by HPLC. The HPLC chromatograms of Spd and Spm in RKO and HCT116 cells are shown in Figure 8A,E. As shown in Figure 8B–D, API at 40 μM significantly reduced the content of Spd, increased the content of Spm, and significantly reduced the ratio of Spd/Spm in RKO cells. Similarly, as shown in Figure 8F–H, API exerted the same regulatory effects on Spd and Spm contents and their ratio in HCT116 cells. The above results validate that API modulates polyamine metabolism by targeting SMOX.

### 2.8. HIF-1α Transcriptionally Regulates and Physically Interacts with SMOX in CRC Cells

**Figure 9:**
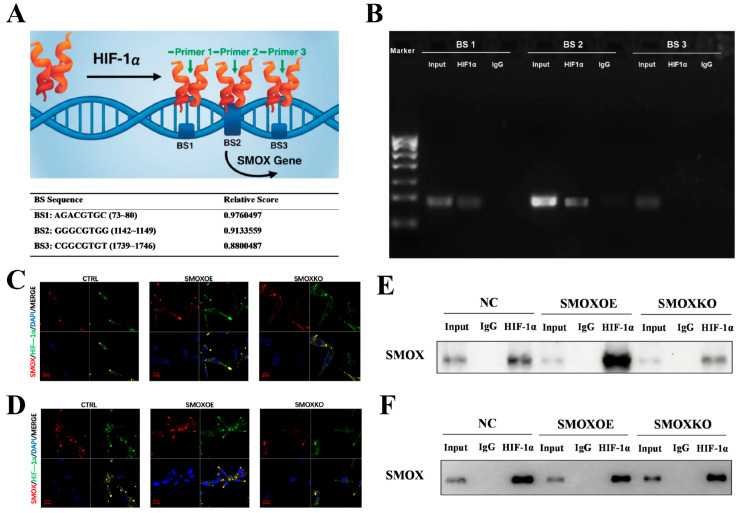
HIF-1α directly binds to the SMOX promoter and physically interacts with the SMOX protein. (**A**) Schematic representation of the SMOX gene promoter containing three putative HIF-1α binding sites (BS1, BS2, and BS3) predicted by the JASPAR database, along with their corresponding relative predictive scores. (**B**) Chromatin immunoprecipitation (ChIP) assay followed by PCR confirming the direct binding of HIF-1α to the predicted sites on the SMOX promoter. (**C**,**D**) Representative immunofluorescence (IF) images showing the co-localization of SMOX (red) and HIF-1α (green) in (**C**) RKO and (**D**) HCT116 cells under negative control (CTRL/NC), SMOX overexpression (SMOXOE), and SMOX knockout (SMOXKO) conditions. Nuclei were stained with DAPI (blue). Scale bar = 10 μm. (**E**,**F**) Co-immunoprecipitation (Co-IP) assays demonstrating the direct physical interaction between HIF-1α and SMOX proteins in (**E**) RKO and (**F**) HCT116 cells across different SMOX expression modification groups.

To profoundly elucidate the intrinsic mechanistic link between these two crucial targets, we investigated their regulatory relationship. Sequence analysis using the JASPAR database predicted three putative HIF-1α binding sites (BS1, BS2, and BS3) on the SMOX promoter with high predictive scores (Figure 9A). A chromatin immunoprecipitation (ChIP) assay confirmed that HIF-1α directly binds to the SMOX promoter, with the most prominent enrichment observed at the BS2 region (Figure 9B), confirming the transcriptional regulation of SMOX by HIF-1α.

Furthermore, we explored their potential protein-level interactions. Immunofluorescence (IF) staining illustrated a robust co-localization of SMOX and HIF-1α within the cells. Notably, the manipulation of SMOX expression—either through overexpression (SMOXOE) or knockout (SMOXKO)—altered the overall expression and spatial distribution patterns of both proteins in RKO and HCT116 cells (Figure 9C,D). The results of co-immunoprecipitation (Co-IP) assays demonstrated that HIF-1α specifically pulled down the SMOX protein, verifying a direct protein–protein interaction. Expectedly, this interaction was markedly amplified in the SMOXOE group and almost abolished in the SMOXKO group (Figure 9E,F). Together, these findings reveal a dual regulatory axis where HIF-1α not only transcriptionally targets the SMOX gene but also physically interacts with the SMOX protein. Coupled with our previous observation that SMOX drives the TLR4/MyD88/HIF-1α inflammatory cascade, these results delineate a vicious “positive feedback loop” in the CRC microenvironment, which is effectively dismantled by API.

## 3. Discussion

CRC is the third most common cancer in the world, and inflammation is an important risk factor for its occurrence and progression [25]. Chronic inflammation is considered one of the key susceptibility factors for CRC in patients with inflammatory bowel disease, which can promote the occurrence and development of the disease. The diagnosis and treatment of CRC have made great progress, but many patients still have the risk of postoperative recurrence and metastasis [26]. To systematically explore these molecular characteristics and develop novel interventions, we utilized untargeted metabolomics and network pharmacology. Our metabolomics analysis revealed significant alterations in inflammation-related and central carbon metabolism pathways in CRC. Network pharmacology further identified apigenin (API) as a promising candidate targeting the HIF-1 signaling pathway and serine/threonine kinase activities, predictions we successfully validated in vitro and in vivo.

To pinpoint the exact metabolic node regulated by API, we pioneeringly employed a high-throughput human endogenous metabolite library screening. Astonishingly, co-incubation with 860 metabolites revealed that polyamine-related metabolites best rescued CRC cells from API treatment. This unbiased screening directly anchored our focus on polyamine metabolism. Polyamines have a variety of functions in cells and can be converted to each other under the action of polyamine oxidase [27]. Polyamines and their metabolites have been studied as cancer biomarkers [14]. However, the metabolism of spermine in CRC has not been fully studied. SMOX plays an important role in promoting key carcinogenic processes and is highly expressed in some types of cancer [28]. Due to the high level of SMOX leading to polyamine-driven oxidative stress and abnormal activation of tumor development, it is necessary to develop polyamine analogues that can selectively inhibit SMOX [29]. These inhibitors can be valuable probes for studying carcinogenic pathways and can be used as chemo-preventive agents [30].

Tumor microenvironments, particularly inflammation and hypoxia, severely disrupt polyamine levels [31]. Traditionally, SMOX-mediated polyamine catabolism generates reactive oxygen species (ROS), which induce DNA damage and activate downstream inflammatory cascades like TLR4/MyD88 [32,33,34]. Strikingly, our study uncovered a novel dual regulatory axis between HIF-1α and SMOX in CRC that fuels this inflammatory network. Through JASPAR database prediction and ChIP assays, we confirmed that HIF-1α acts as a transcription factor directly binding to the SMOX promoter. More profoundly, Co-IP and immunofluorescence assays revealed a direct physical interaction between HIF-1α and SMOX proteins, establishing a complex that exacerbates metabolic dysregulation. API exerts its potent anti-CRC effects by acting as a “circuit breaker”—profoundly dismantling this HIF-1α/SMOX transcriptional and post-translational interaction axis, thereby downregulating the downstream TLR4/MyD88 and MAPK/ERK inflammatory cascades.

Consistent with these molecular findings, our HPLC analysis demonstrated that API reshapes intracellular polyamine metabolism. The ratio of Spd/Spm is a direct indicator of SMOX activity [35]. API effectively reduced Spd levels, increased Spm levels, and decreased the Spd/Spm ratio in CRC cells. This biochemical phenotype aligns perfectly with our metabolite library screening results, confirming that API therapeutically targets polyamine metabolism.

However, this study has certain limitations. While our findings emphasize that API functions by modulating the TLR4/MyD88 inflammatory cascade and the tumor microenvironment, the in vivo validation was performed using BALB/c nude mice. Because these mice are immunodeficient and lack functional T-cells, our current model cannot fully capture the complex interactions between API and the adaptive immune system. Future studies employing immunocompetent murine models will be necessary to comprehensively elucidate the immunomodulatory role of API and the HIF-1α/SMOX axis within an intact immune microenvironment.

In summary, our findings introduce a paradigm-shifting approach by integrating multi-omics with high-throughput metabolite screening. The identification of the API-targeted HIF-1α/SMOX dual regulatory axis provides a solid theoretical foundation and novel therapeutic targets for precision CRC treatment using natural metabolic modulators [36,37,38].

## 4. Materials and Methods

### 4.1. Untargeted Metabolomics Analysis

To investigate the metabolic profiles of colorectal cancer, untargeted metabolomics was performed on paired tumors and adjacent normal tissues. A total of 11 tissue specimens were collected from patients with rectal adenocarcinoma who underwent surgical treatment at the Department of Gastrointestinal Surgery, Affiliated Hospital of North Sichuan Medical College, between June and July 2021. Paired adjacent normal tissues were taken >5 cm from the tumor margin. Informed consent was obtained from all patients, and the study was approved by the hospital’s Ethics Committee.

Briefly, tissue samples were homogenized with steel beads (50 Hz, 60 s) in an extraction solvent containing 75% methanol-chloroform (9:1, *v*/*v*) and 25% H_2_O. Following sonication and centrifugation (12,000 rpm, 4 °C for 10 min), the supernatants were collected, dried, and reconstituted in 50% acetonitrile containing 2-chlorophenylalanine (4 ppm) as an internal standard. The metabolic profiling was conducted using a Waters ACQUITY UPLC system (Waters Corporation, Milford, MA, USA) equipped with an ACQUITY UPLC^®^ HSS T3 column (Waters Corporation, Milford, MA, USA) (2.1 × 150 mm, 1.8 μm) coupled to a Thermo Q Exactive mass spectrometer (Thermo Fisher Scientific, Waltham, MA, USA) operating in both positive and negative ESI modes.

Raw MS data were converted to mzXML format using ProteoWizard MSConvert (Version 3.0.3, ProteoWizard Software Foundation, Palo Alto, CA, USA) and processed with the R XCMS package (Version 3.6.1, R Foundation for Statistical Computing, Vienna, Austria) for peak picking, alignment, and filtering. Metabolite annotation was performed by matching against public databases, including HMDB, LipidMaps, mzCloud, and KEGG. Data normalization was achieved via the LOESS signal correction method based on quality control (QC) samples. Multivariate statistical analyses, including PCA, PLS-DA, and OPLS-DA, were performed using R software. Differential metabolites were strictly defined by a Variable Importance in Projection (VIP) score > 1 and a *p*-value < 0.05. Finally, functional pathway enrichment and topological analysis of these differential metabolites were conducted using MetaboAnalyst (Version 5.0, Xia Lab, McGill University, Montreal, QC, Canada) and visualized via KEGG Mapper (Kanehisa Laboratories, Kyoto, Japan).

### 4.2. Network Pharmacology Analysis

The 2D structure and canonical SMILES of apigenin (API) were retrieved from the PubChem database. Potential targets of API were predicted using the SwissTargetPrediction database (limited to the species Homo sapiens) and the Traditional Chinese Medicine Systems Pharmacology (TCMSP) database. Colorectal-cancer-related targets were comprehensively collected from the GeneCards and OMIM databases using the keyword “colorectal cancer”. To ensure data reliability, disease targets were filtered based on a relevance score greater than the median. The intersection of API targets and CRC-related targets was obtained and visualized using a Venn diagram.

A protein–protein interaction (PPI) network of these intersecting targets was constructed using the STRING database, with the species explicitly set to Homo sapiens and a minimum required interaction score > 0.400 (medium confidence). The resulting PPI network was imported into Cytoscape software (Version 3.9.1, Cytoscape Consortium, San Diego, CA, USA) for visualization and topological analysis. Core hub genes (such as *HIF1A, MYC,* and *TP53*) were identified using the cytoHubba plugin ytoHubba plugin (Version 0.1, Cytoscape Consortium, San Diego, CA, USA) based on degree centrality. Finally, Gene Ontology (GO) and Kyoto Encyclopedia of Genes and Genomes (KEGG) pathway enrichment analyses of the core targets were performed using the “clusterProfiler” and “org.Hs.eg.db” packages in R software (Version 3.6.3, R Foundation for Statistical Computing, Vienna, Austria). The enrichment significance thresholds were strictly set at a *p*-value < 0.05 and a False Discovery Rate (FDR) < 0.05 to identify significantly enriched pathways.

### 4.3. Molecular Docking Analysis

UCSF Chimera (Version 1.16, Resource for Biocomputing, Visualization, and Informatics, University of California, San Francisco, CA, USA) and Autodock Vina (Version 1.2.3, Center for Computational Structural Biology, Scripps Research Institute, La Jolla, CA, USA) were employed to analyze the binding affinities and modes of interaction between the drug candidate and their targets. The Canonical SMILES of API was retrieved from PubChem Compound. The 3D coordinates of SMOX (PDB ID, 7OY0; resolution, 2.09 Å) were downloaded from the PDB. For docking analysis, all protein and molecular files were converted into PDB format with all water molecules excluded and polar hydrogen atoms were added using UCSF Chimera 1.16. The grid box was centered to cover the domain of the protein and accommodating free molecular movement when using Autodock Vina 1.2.3 for molecular docking. The docked conformation with hydrogen bond and low docked energy was selected and visualized by UCSF Chimera 1.16.

### 4.4. Cell Culture and Viability Assay

HCT116 and RKO cells were purchased from Jiangsu KeyGEN BioTECH Corp (Nanjing, China). The cells were cultured at 37 °C and 5% CO_2_ in DMEM Medium 1640 (Gibco, Thermo Fisher Scientific, Waltham, MA, USA) that contained 10% fetal bovine serum (Gibco, Thermo Fisher Scientific, Waltham, MA, USA) and 1% penicillin–streptomycin (Gibco, Thermo Fisher Scientific, Waltham, MA, USA). The cell counting kit 8 (CCK 8) assay (APE×BIO Technology LLC, Houston, TX, USA) was used to determine cell viability. Briefly, cells were treated with different concentrations of API (J&K Scientific, Beijing, China) for 24 h, and 10% CCK-8 solution was added to each well. After incubation at 37 °C for 2 h, the absorbance was read at 490 nm on the microplate reader (SpectraMax 190, San Jose, CA, USA).

### 4.5. Human Endogenous Metabolite Library Screening

To identify the specific metabolic pathways regulated by API, a high-throughput screening was conducted using the Human Endogenous Metabolite Library (MCE HY-L030, MedChemExpress, Monmouth Junction, NJ, USA), which contains 860 well-characterized human endogenous metabolites. RKO and HCT116 cells were seeded in 96-well plates. Cells were co-incubated with API 40 μM and individual metabolites from the library for 24 h. Cell viability was evaluated using the CCK-8 assay. Cell viability was evaluated using the CCK-8 assay. The relative viability was calculated as the ratio of the absorbance in the (API + Metabolite) group to that in the (API + DMSO) control group.

To systematically isolate robust biological signals from the minor background fluctuations inherent to high-throughput screening, an empirical dual threshold was established based on the assay’s distribution profile. Specifically, candidate metabolites were strictly defined as significant “hits” only if they achieved a relative viability ratio ≥ 1.20 (representing a robust > 20% restoration of cell viability, effectively separating them from background noise) combined with statistical significance (*p* < 0.05, evaluated by independent Student’s *t*-test). Metabolites meeting these stringent criteria—predominantly polyamine-related molecules such as spermine, spermidine, and arginine—were subsequently selected for in-depth mechanistic validation.

### 4.6. Cell Scratch, Invasion and Apoptosis Assays

For cell scratch assay, colorectal cancer cells in logarithmic growth phase were made into cell suspension, and the cell density was adjusted to 1 × 10^5^/mL and inoculated into 6-well culture plates. The cells with uniform and more than 90% fusion were taken, and the cells were evenly crossed with 200 μL sterile gunhead perpendicular to the horizontal line. After scribing, the suspension cells were washed with PBS, and different concentrations of drug-containing and serum-free medium were added. The migration of cells was observed by microscope at 0 h, 24 h and 48 h, and the scratch width of cells was recorded at the same position.

For cell invasion assay, colorectal cancer cells in logarithmic growth phase were starved for 6 h in serum-free medium. After 1 μL Matrigel was mixed with 49 μL serum-free medium, the upper chamber membrane of a transwell chamber was added and placed in an incubator at 37 °C for 3 h to agglomerate Matrigel. A total of 600 μL medium containing 20% serum was added to the lower chamber. The cells were digested with trypsin, the cells were washed with PBS to remove serum, and the cells were resuspended in serum-free medium to count and adjust the cell density to 5 × 10^4^/mL. A total of 300 μL cell suspension was inoculated into the transwell upper chamber containing Matrigel glue, and different concentrations of the test substance were added to make the cells evenly spread in the upper chamber for 24 h. After removing from the chamber, they were washed with PBS 3 times and a cotton swab was used to wipe residual cells and matrix glue. The chamber was immersed in methanol solution for 15 min; then the chamber was immersed in 0.1% crystal violet dye solution dissolved in methanol for 15 min, washed three times with PBS, the purple color on the membrane washed off, and dried in an oven at 37 °C. The cell penetration was observed by microscope, photographed and counted.

Apoptosis was detected by Annexin V FITC and PI double staining. The cells treated with the test drugs in the 6-well plate were operated according to the kit instructions (Jiangsu KeyGEN Bio TECH Corp., Ltd., Nanjing, China). At the same time, the medium and trypsin-digested cells were centrifuged to remove the supernatant, washed with PBS and centrifuged to collect cells. The mixed staining solution of Annexin V FITC and PI was added according to 250 μL 1 × Binding Buffer, 2.5 μL Annexin V FITC and 2.5 μL PI Staining Solution per well and incubated at room temperature for 15 min in the dark. Apoptosis was detected by flow cytometry.

### 4.7. Animals

SPF male BALB/c nu/nu mice, weighing 18–22 g, were purchased from Chengdu Dashuo Laboratory Animal Technology Co., Ltd.(Chengdu, China). With a total of 8 mice used, with *n* = 4 per group, they were housed in an animal laboratory at 25 °C and 60% humidity. A total of 2 × 10^6^ RKO cells were inoculated subcutaneously into the back of nude mice. After 2 weeks of tumor formation in nude mice, qualified nude mice were randomly divided into 2 groups: control (CTR) and API (50 mg/kg). In this study, a single animal was considered as an experimental unit. After 21 days (q.d.) of API intervention, the nude mice were sacrificed, and the back subcutaneous tumors were isolated. The tumor tissue was used for subsequent experimental studies.

All experimental protocols and animal handling procedures were approved by the Institutional Review Board of West China Hospital Experimental Animal Ethics Committee (protocol code 20230925002).

### 4.8. Histological Staining

Histological staining followed the standard HE staining procedure. The tumor tissue was fixed with 4% paraformaldehyde solution for 24 h. After alcohol gradient dehydration, it was embedded in transparent paraffin, cut into about 3 μm thick coronal sections, dewaxed with xylene and alcohol dehydration was conducted. Each tissue section was baked at 60 °C for 24 h and xylene Ⅰ and Ⅱ dewaxing, decreasing concentration gradient alcohol dehydration, hematoxylin staining, 1% hydrochloric acid alcohol differentiation, water flushing back to blue, 5% Eosin counterstaining, 80%, 95%, and 100% alcohol dehydration, xylene transparency, and neutral gum seal were performed. Microscope observation and image acquisition analysis were conducted.

### 4.9. Immunohistochemistry

The tumor tissue was fixed with 4% paraformaldehyde solution for 24 h. All tissues were embedded in paraffin and sectioned. The steps of embedding and slicing were performed as described in histological staining. The SMOX primary antibodies (1:500; Protein Tech, Rosemont, IL, USA) were used to detect the expression of SMOX in tumor tissues.

### 4.10. Quantitative Real-Time PCR Analysis

Quantitative real-time PCR (qRT-PCR) analysis was performed as described in a previous study [39]. Briefly, after the animals were treated with API, the total RNA of each group was extracted, and the RNA was reversely transcribed into cDNA. The expression of SMOX gene in each group was detected by qRT-PCR, which was conducted using a qTOWER3 G system (Analytikjena, Jena, Germany) to determine the mRNA expression of the gene of interest. Expression levels were normalized to GAPDH. The results were analyzed using the 2^−ΔΔCt^ method for relative quantification. The list of primers designed is shown in Table 1.

### 4.11. Western Blot Analysis

Western blotting was performed as described in a previous study [39,40]. Briefly, after animals or cells were treated with apigenin, tumor tissues or cells were lysed with RIPA lysate to extract proteins. Protein concentration was determined by BCA kit (Beyotime Biotechnology, Shanghai, China). After electrophoresis, the protein sample was transferred to polyvinylidene fluoride membrane, blocked with 5% skim milk for 1 h, and incubated with related primary antibodies. The following antibodies were used: SMOX (1:1000), TLR4 (1:1000), MyD88 (1:1000), phosphor (p)-p38 MAPK (1:500), p38 MAPK (1:1000), phosphor (p)-ERK1/2 (1:500), ERK1/2 (1:1000), phosphor (p)-JNK (1:500), JNK (1:1000), TNFα (1:1000), IL 1β (1:1000), HIF 1α (1:1000) and β-actin (1:1000) (all from Protein Tech, Rosemont, IL, USA). After the first antibody incubation, the appropriate second antibody was selected for incubation, and the chemiluminescence kit (Beyotime Biotechnology, Shanghai, China).was used to image through the gel imaging system. The relative gray value of the signal is obtained by Quantity one (Version 4.6.2, Bio-Rad Laboratories, Hercules, CA, USA).

### 4.12. HPLC Analysis

High-performance liquid chromatography (HPLC, LC-2030C Plus, Shimadzu Corporation, Kyoto, Japan) was used to measure the content of Spm and Spd in RKO and HCT116 cells. After the cells were treated with API, 3 mL of 5% pre-cooled perchloric acid was added, incubated on ice for 1 h at 12,000 r/min and 4 °C, centrifuged for 30 min, and 2 mL of supernatant was collected for derivatization. The filtrate after derivatization was used for liquid chromatography detection. The chromatographic conditions of Spd and Spm were as follows: C18 column (5 μm C18, 4.6 × 250 mm), column temperature 25 °C, mobile phase (methanol/water, 53:47), flow rate 0.5 mL/min, injection volume 50 μL, and detection wavelength 234 nm. The chromatograms were recorded and analyzed by Labsolutions system software (Version 5.97, Shimadzu Corporation, Kyoto, Japan).

### 4.13. Chromatin Immunoprecipitation (ChIP) Assay

The putative transcription factor binding sites of HIF-1α on the SMOX promoter were predicted using the JASPAR database (https://jaspar.genereg.net (accessed on 15 October 2025)). The ChIP assay was performed using a ChIP assay kit (Protein Tech, Rosemont, IL, USA) according to the manufacturer’s instructions. Briefly, cells were cross-linked with 1% formaldehyde and quenched with glycine. After cell lysis, chromatin was sonicated to shear DNA into fragments of 200–500 bp. The sheared chromatin was immunoprecipitated overnight at 4 °C with an anti-HIF-1α antibody or normal IgG (as a negative control). The cross-linking was reversed, and the precipitated DNA was purified and analyzed by PCR using primers specifically designed for the predicted SMOX promoter binding sites.

### 4.14. Immunofluorescence (IF) Staining

Cells cultured on glass coverslips were fixed with 4% paraformaldehyde, permeabilized with 0.5% Triton X-100, and blocked with 5% BSA. The cells were then incubated overnight at 4 °C with primary antibodies against SMOX and HIF-1α. After washing, cells were incubated with corresponding fluorescently labeled secondary antibodies for 1 h at room temperature in the dark. Nuclei were counterstained with DAPI. Images were acquired using a confocal laser scanning microscope (Carl Zeiss AG, Oberkochen, Germany).

### 4.15. Co-Immunoprecipitation (Co-IP) Assay

To detect the protein–protein interaction between HIF-1α and SMOX, cells were lysed using a weakly denaturing IP lysis buffer supplemented with protease inhibitors. Equal amounts of total protein lysates were incubated overnight at 4 °C with anti-HIF-1α primary antibody or control IgG. Subsequently, protein A/G magnetic beads were added and incubated for 2 h to capture the immune complexes. The beads were washed extensively, and the bound proteins were eluted by boiling in SDS loading buffer. The eluents, along with the input samples, were then subjected to Western blot analysis to detect the presence of SMOX.

### 4.16. Statistical Analysis

Statistical analysis was performed using GraphPad Prism (Version 9.0, GraphPad Software, San Diego, CA, USA). The experimental data were expressed as mean ± standard error of the mean (SEM). Prior to comparative analysis, the Shapiro–Wilk test was used to verify the normal distribution of the data. For normally distributed data, the Student’s *t*-test was used for the comparison between the two groups, and the one-way analysis of variance was used for the comparison between more than two groups. The LSD method was used for the homogeneity of variance, and the Tamhane’s method was used for the heterogeneity of variance. The levels of significance were set at * *p* < 0.05 and ** *p* < 0.01.

## 5. Conclusions

**Figure 10:**
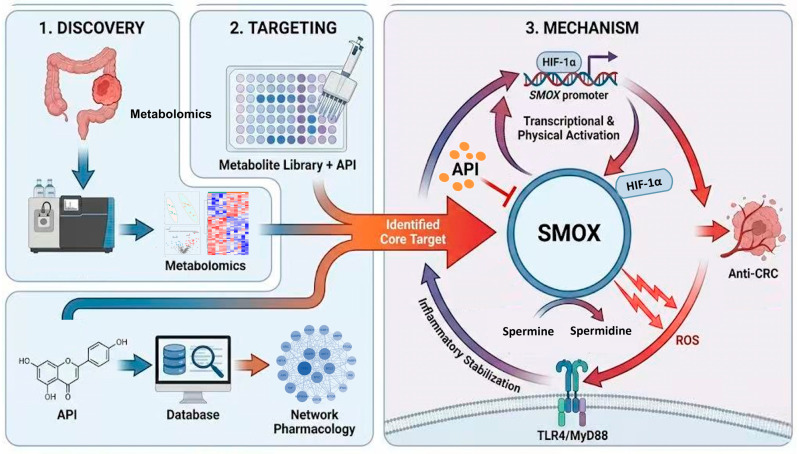
Schematic representation of the discovery strategy and the underlying mechanism of apigenin (API) against colorectal cancer.

In this study, by integrating untargeted metabolomics, network pharmacology, and a pioneering high-throughput endogenous metabolite library screening, we successfully identified polyamine metabolism and SMOX as the precise therapeutic vulnerabilities targeted by API in CRC. API exhibited potent anti-CRC efficacy both in vitro and in vivo. Mechanistically, we uncovered a novel vicious cycle: HIF-1α/SMOX positive feedback loop, where HIF-1α transcriptionally and physically upregulates SMOX, while SMOX feeds back to stabilize HIF-1α via the TLR4/MyD88 inflammatory cascade. API acts as a powerful “circuit breaker” to dismantle this loop (Figure 10). These findings provide a discovery-driven paradigm and a solid theoretical foundation for developing API as a targeted metabolic modulator in CRC therapy.

## Figures and Tables

**Table 1:** List of primers designed for qRT-PCR.

Primer Name	Primer Sequence	
SMOX	Forward	5′-ACGGAGATGCTGCGTCAGTTCA-3′
	Reverse	5′-CCTGCGTGTATGAATAGGAGCC-3′
GAPDH	Forward	5′-GTCTCCTCTGACTTCAACAGCG-3′
	Reverse	5′-ACCACCCTGTTGCTGTAGCCAA-3′

## Data Availability

The raw data supporting the conclusions of this article will be made available by the authors on request.

## Supplementary Materials

The following supporting information can be downloaded at: https://www.mdpi.com/article/10.3390/ijms27073261/s1.
