# Supplementary material for: Targeting Polyamine Metabolism in Colorectal Cancer: Apigenin Dismantles the HIF-1α/SMOX Positive Feedback Loop to Suppress Tumor Progression
Source: Int J Mol Sci. 2026 Apr 3;27(7):3261. doi: 10.3390/ijms27073261 (PMC13074065; doi:10.3390/ijms27073261)
Supplement: Supplementary file 1 [file ijms-27-03261-s001.zip › ijms-4196867-supplementary.pdf]

Supplementary Material

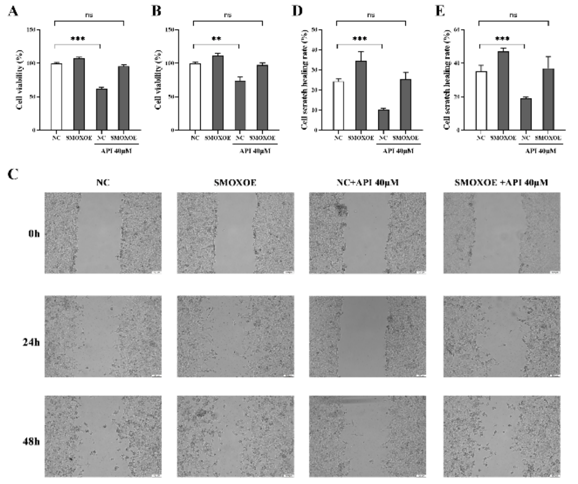

Supplementary Figure 1. Overexpression of SMOX rescues colorectal cancer cells from API-induced anti-proliferative and anti-migratory effects. (A, B) Cell viability of (A) RKO and (B) HCT116 cells transfected with empty vector (NC) or SMOX overexpression plasmids (SMOXOE) and subsequently treated with or without 40 μM API for 24 h. Cell viability was evaluated using the CCK-8 assay. (C) Representative microscopic images of the cell scratch assay at 0, 24, and 48 h of RKO cells. Scale bar = 100 μm. (D, E) Quantitative analysis of the cell scratch healing rate in (D) 24h and (E) 48h across the indicated treatment groups. Data are presented as mean ± SEM. ** $p < 0.01$, *** $p < 0.001$; ns, not significant.
